# HybridGCN for protein solubility prediction with adaptive weighting of multiple features

**DOI:** 10.1186/s13321-023-00788-8

**Published:** 2023-12-08

**Authors:** Long Chen, Rining Wu, Feixiang Zhou, Huifeng Zhang, Jian K. Liu

**Affiliations:** 1 Readline Intelligence, Leeds, United Kingdom; 2 School of Computing, University of Leeds, Leeds, United Kingdom; 3 School of Computer Science, University of Birmingham, Birmingham, United Kingdom

**Keywords:** Protein solubility prediction, ESM, Zero-shot learning, Feature fusion, Adaptive feature reweighting

## Abstract

The solubility of proteins stands as a pivotal factor in the realm of pharmaceutical research and production. Addressing the imperative to enhance production efficiency and curtail experimental costs, the demand arises for computational models adept at accurately predicting solubility based on provided datasets. Prior investigations have leveraged deep learning models and feature engineering techniques to distill features from raw protein sequences for solubility prediction. However, these methodologies have not thoroughly delved into the interdependencies among features or their respective magnitudes of significance. This study introduces HybridGCN, a pioneering Hybrid Graph Convolutional Network that elevates solubility prediction accuracy through the combination of diverse features, encompassing sophisticated deep-learning features and classical biophysical features. An exploration into the intricate interplay between deep-learning features and biophysical features revealed that specific biophysical attributes, notably evolutionary features, complement features extracted by advanced deep-learning models. Augmenting the model’s capability for feature representation, we employed ESM, a substantial protein language model, to derive a zero-shot learning feature capturing comprehensive and pertinent information concerning protein functions and structures. Furthermore, we proposed a novel feature fusion module termed Adaptive Feature Re-weighting (AFR) to integrate multiple features, thereby enabling the fine-tuning of feature importance. Ablation experiments and comparative analyses attest to the efficacy of the HybridGCN approach, culminating in state-of-the-art performances on the public eSOL and S. cerevisiae datasets.

## Introduction

Protein solubility is a critical biophysical characteristic that is essential for evaluating the effectiveness of proteins in biological and chemical engineering. It is a major factor in pharmaceutical research and production yield. Poor solubility of proteins can impede protein production, leading to the development of various strategies to improve it, such as using low temperatures [1], weak promoters [2], and optimizing growth media [3]. The primary structure of proteins, particularly the amino acid sequence, is a major determinant of protein solubility. Studies [4, 5] have shown a strong correlation between protein solubility and sequence-based features, such as the presence of hydrophobic stretches, the composition of different residue types, and the length of the protein sequence. As a result, prediction techniques that use sequence-based information to estimate solubility [6, 7] have gained considerable attention in the protein engineering research community. These techniques offer the potential to replace expensive experimental procedures by pre-selecting the most promising protein sequences.

Numerous machine learning-based prediction methods have been developed to estimate protein solubility using sequence-based information. These methods employ models such as Support Vector Machines (SVM) [8], Naive Bayes [6], and Neural Networks [9], as well as hand-crafted features that encompass structural and biological characteristics. By optimizing the design of various bioprocesses, machine learning models have significantly improved solubility prediction accuracy. However, most existing machine learning models [10, 11] are trained for binary classification tasks, categorizing datasets into soluble and insoluble categories, rather than providing continuous solubility values, which are more desirable. In the field of protein engineering, continuous solubility values are more significant than binary classifications, as they offer more informative guidance for downstream tasks. For instance, in large protein datasets, the selection of optimal protein sequences can be performed based on continuous solubility values, while binary values fall short in accomplishing this task. Moreover, traditional machine learning models have recently fallen behind deep learning models in terms of performance due to the limited generalization capacity of handcrafted features. Deep learning models have achieved the state-of-the-art (SOTA) performance on various protein engineering tasks, including structure prediction [12, 13], protein design [14, 15], protein binder design [16], stability prediction [17, 18], and solubility prediction [9, 19]. Therefore, it is important to understand how to combine different biophysical and deep-learning features into one single model yet in a more flexible fashion.

In this investigation, we explore the nuanced interplay between deep features and classical manually curated features, with a particular focus on the complementary role played by specific classical features, notably evolutionary features. Grounded in this insightful observation, we introduce HybridGCN, an innovative hybrid graph convolutional network meticulously designed to harness the synergies between manually crafted features and advanced deep features. This integration yields notable advancements in the accuracy of predicting protein solubility. Embedded within the HybridGCN framework is the novel Adaptive Feature Re-weighting (AFR) module, which orchestrates the seamless fusion of domain-specific knowledge encapsulated in handcrafted features with the discriminative insights extracted from high-capacity deep learning models. The AFR module serves as an intelligent mechanism for recalibrating feature importance, ensuring a refined and contextually informed representation of the input features in the prediction process. Furthermore, to bolster the feature representation capacity of HybridGCN, we introduce the ESM-1v feature, derived from zero-shot learning. This feature proves instrumental in capturing expansive and relevant information pertaining to protein functions and structures, thereby enriching the predictive capabilities of the model in the domain of protein solubility. The proposed HybridGCN framework, incorporating a blend of handcrafted, deep, and zero-shot learning features, stands as a significant contribution to the field, showcasing a comprehensive approach towards advancing predictive models for protein solubility.

The key contributions of our work can be summarized as follows:

- We present a novel graph convolutional network, HybridGCN, which effectively merges advanced deep features and classic solubility-related features, resulting in a significant improvement in protein solubility prediction performance.
- We explore the interrelations between deep features and classic features, revealing their complementary nature. Furthermore, we introduce the ESM-1v feature, a zero-shot learning feature, to enhance the input features of HybridGCN. The inclusion of the ESM-1v feature enables the capture of comprehensive information relevant to protein functions and structures, thereby benefiting the protein solubility prediction task.
- We propose an AFR module that dynamically adjusts the importance of different features, prioritizing the most informative ones for the solubility prediction task.
- We conduct extensive ablation experiments and comparison experiments to validate the effectiveness of the ESM-1v feature and the AFR module. The results demonstrate that the proposed HybridGCN achieves SOTA performance on the publicly available eSOL dataset.

The rest of the paper is organized as follows. Section Related work summarizes the related works. Section The proposed hybridGCN describes the proposed HybridGCN. Section Experimental setup describes the experimental set-up and Sect. Results and discussion reports the experimental results and discussions.

## Related work

In recent years, the application of machine learning (ML), particularly deep learning, in protein solubility prediction has gained significant attention. Many ML-based solubility prediction methods have been developed and published, broadly categorized into traditional machine learning-based methods and deep learning-based methods.

### Models based on traditional ML methods

Traditional machine learning models have been extensively utilized for classification and regression tasks, including protein solubility prediction. Several sequence-based machine learning methods have been developed in this context, such as PaRSnIP [5], PROSO II [11], CCSOL [20], SOLpro [21], PROSO [6], RPSP [10], and the scoring card method (SCM) [22]. These methods share a common approach of extracting handcrafted features from protein sequences based on domain knowledge in bioinformatics. These features are then used as input for downstream classifiers or regressors to accomplish protein solubility prediction tasks.

Among the ML-based methods, support vector machine (SVM) [23] is a commonly employed model for distinguishing between soluble and insoluble proteins. Idicula et al. [8] proposed an SVM classifier for this purpose and demonstrated its potential in identifying soluble protein variants during the screening of protein libraries. Agostini et al. [20] developed a webserver called ccSOL, which utilizes an SVM classifier along with several biological features (e.g., coil/disorder, hydrophobicity, $\beta$-sheet, and $\alpha$-helix propensities) to predict solubility for endogenous and heterologous expression in Escherichia coli. Validation on three independent sets showed that ccSOL achieved an accuracy of 74% on 31,760 protein sequences for discriminating soluble and insoluble proteins. To enhance the accuracy of protein solubility prediction, Magan et al. [21] proposed a two-stage SVM approach in which the first stage selects 20 out of 23 features and trains 20 independent SVMs, while the second stage trains a single SVM using the ensemble of selected features. This ensemble strategy significantly improved accuracy. Similarly, the PROSO method introduced in [6] also employs a two-stage classifier for solubility prediction. The first stage, a primary SVM classifier, focuses on feature selection, and its outputs serve as inputs for the second Naive Bayes classifier. PROSO outperforms previously reported solubility predictors and identifies the subset of features that have the strongest impact on protein solubility.

In addition to SVM, various other ML models have been employed for protein solubility prediction. For instance, RPSP [10] performs classification using a standard Gaussian distribution to distinguish soluble proteins from insoluble ones, while SCM [22] employs a scoring card approach, utilizing only dipeptide composition to estimate the solubility scores of protein sequences. PROSO II [11] constructs a two-stage classifier consisting of a Parzen window model and two logistic regression classifiers. The outputs of the primary Parzen window model and logistic regression classifier serve as inputs for the logistic regression classifier in the second stage. The PaRSnIP [5] adopts a nonlinear predictive model called gradient boosting machine (GBM) for protein solubility prediction. Compared to the black-box model SVM, GBM offers the advantage of identifying the properties of protein sequences that contribute most to distinguishing between soluble and insoluble protein sequences.

### Deep learning models

Deep learning has demonstrated remarkable success in various domains, including natural language processing [24], image classification [25], and protein engineering [26, 27]. Unlike most previous two-stage machine learning methods, deep learning-based approaches have the advantage of automatically extracting discriminative features from raw data without the need for explicit feature selection. For instance, Khurana et al. [9] introduced DeepSol, a convolutional neural network (CNN), to extract discriminative features directly from raw protein sequences for protein solubility prediction. DeepSol aims to classify protein sequences as either soluble or insoluble, and it incorporates additional biological and structural features to enhance the deep features, resulting in improved classification accuracy. These findings highlight the complementarity of biological and structural features with deep features. Similarly, EPSOL [28] utilizes a shallow CNN to process raw sequences along with other biological and structural features, effectively leveraging multiple features to achieve satisfactory prediction performance.

Given that the performance of deep learning models is highly dependent on the amount of training data, ProGAN [29] introduces a Generative Adversarial Network (GAN) to generate additional data for augmenting the training set, further enhancing the prediction performance of protein solubility. TAPE [30] and SeqVec [7] employ a pre-training strategy on large-scale protein datasets, followed by transfer learning to the downstream solubility prediction task. NetSolP [31] utilizes advanced transformer architecture for protein solubility prediction. RPPSP [32] exploits a novel protein sequence encoder to generate statistical representations of protein sequences that improve prediction accuracy. However, they do not incorporate spatial information from protein sequences.

Graph Convolutional Networks (GCNs) have achieved notable success in protein structure representation and properties prediction. However, GCNs typically require 3D structural information as input, which is often challenging to obtain solely from protein sequences. Fortunately, advanced protein structure prediction methods can generate accurate protein contact maps as substitutes for 3D structures. GraphSol [19] is the first work to construct a protein topology attribute graph using predicted protein contact maps. It employs a graph convolutional network to predict protein solubility, leveraging the power of GCNs in this context.

### Feature engineering

Feature engineering [33], a crucial step in traditional machine learning systems, involves designing and selecting robust features based on domain knowledge [34, 35]. The discriminative power and robustness of these features significantly influence the performance of machine learning models [36]. In the realm of protein solubility prediction, early studies such as [10] explored solubility-related features. They analyzed six sequence-based features, including average charge, turn-forming residue fraction, cysteine fraction, proline fraction, hydrophilicity, and total number of residues, and revealed strong correlations between average charge, turn-forming residue fraction, and protein solubility. Subsequent works further established strong associations between primary sequence characteristics and protein solubility. For instance, Idicula et al. [8] selected physicochemical properties, residue compositions, and dipeptide compositions as features to train a SVM classifier for predicting over-expression status in E. coli. This model achieved an accuracy of approximately 72%, indicating the reasonable performance of the selected features in predicting protein solubility. Similarly, Agostini et al. [20] identified features such as $\alpha$-helix propensities, $\beta$-sheet content, hydrophobicity, and coil/disorder as highly relevant to protein solubility.

In machine learning, the ensemble algorithm is an effective way to obtain better predictive performance [37]. Magan et al. [21] carefully selected multiple kinds of features and trained multiple independent SVM classifiers using these features, finally, they achieved significantly improved prediction accuracy by the ensemble of multiple SVM classifiers. PROSO [6] employed a two-stage classifier for solubility prediction. The first-stage classifier performed feature ranking by measuring the symmetrical uncertainty of attributes with respect to the given class. Notably, the frequencies of dipeptides with the first residue charged and the second non-polar residue emerged as the most important determinants of protein solubility according to the feature ranking results. Furthermore, PROSO II [11] analyzed the significance of features and their correlation with protein solubility, selecting only features that exhibited a significant correlation for predicting protein solubility. In the case of PaRSnIP [5], it exploited 8,477 features for each amino acid sequence, encompassing frequency-based features (e.g., tripeptide frequencies and turn-forming residues) and structural features (e.g., secondary structure and relative solvent accessibility information). PaRSnIP utilized the GBM as the predictive model, which provided feature importance measures for distinguishing between soluble and insoluble protein sequences. Consequently, PaRSnIP did not perform feature selection to exclude features but relied on the GBM to identify and prune non-essential features.

## The proposed hybridGCN

Protein solubility prediction is a regression task, which can be formulated as a mapping function *f* between the input sequence ${\textbf{p}} \in {\mathbb {R}}$ and the solubility value ${\textbf{s}}$, i.e, $f\,\ P\rightarrow S\in [0, 1]$. In this work, we propose a two-stage deep framework for protein solubility prediction, in which the first stage is the extraction of multiple features, including biological features and high-level deep learning features. Then, we propose a GCN that can be seen as a deep predictor. Specifically, We propose a novel graph convolutional network, named HybridGCN, for the protein solubility prediction task focusing on the regression of every value. The graph is the most fundamental part of GCN, which consists of two components: nodes (vertices) and edges. A graph *G* can be defined as *G*(*V*, *E*), where *V* is the set of nodes, and *E* is the set of edges between the nodes. We model the protein sequence data using the graph structure, and propose a graph convolutional network to construct the mapping between protein sequences and the corresponding solubility values, the mapping denoted as *f* can be formulated as $f\,\ G(V,\ E)\rightarrow S$, where *S* denotes the solubility values.

### The overview of hybridGCN

**Fig. 1:**
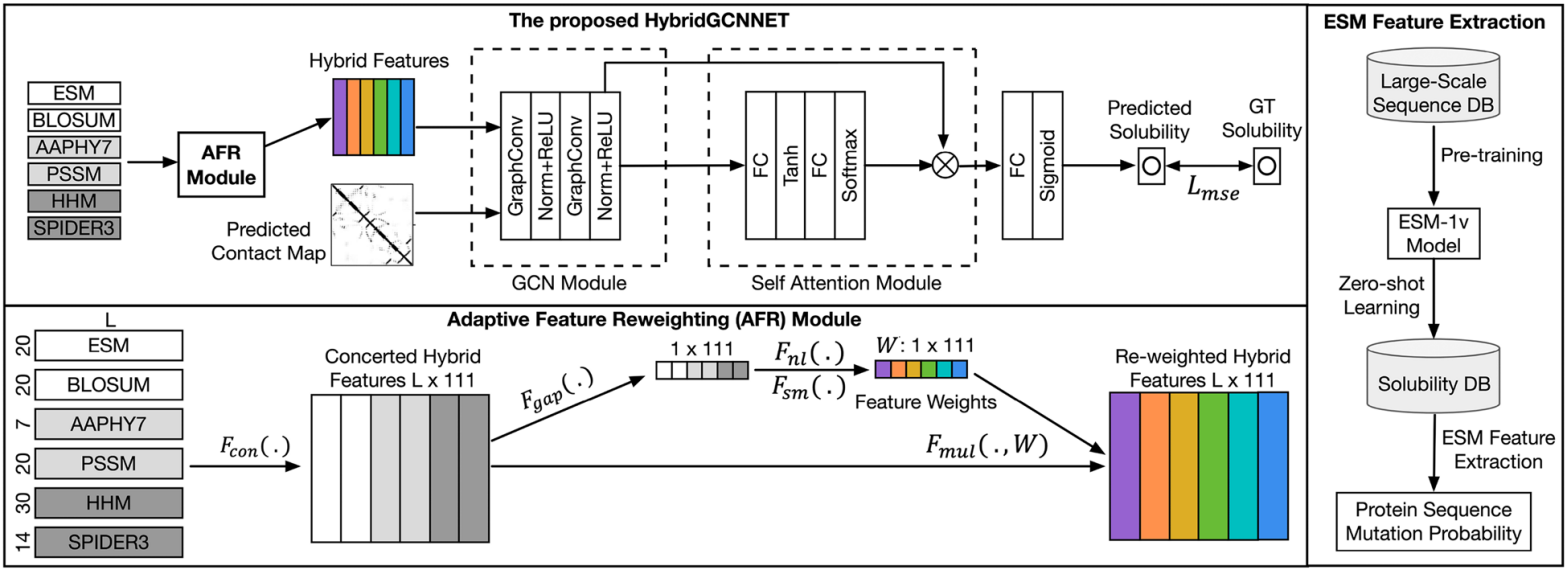
The overview of the proposed HybridGCN and the adaptive feature reweighting (AFR) module

HybridGCN consists of several modules, including the AFR module, the GCN module, and the self-attention module. The AFR module dynamically adjusts the importance of different node features for better performance. The GCN module is mainly to fuse different node features and edge features. The self-attention module enables HybridGCN to focus on learning the most relevant feature channels in the fused features. The self-attention module has two fully connected (FC) layers to extract hidden features. Tanh and softmax activation functions are added after two FC layers to rectify fused features with nonlinearity. The softmax converts the hidden layer into a normalized probability distribution, ensuring that the attention scores sum up to 1 and indicating the relative importance of each hidden node in the hidden layer. Finally, we employ the sigmoid loss function to transform the last hidden layer into a predicted solubility value between 0 and 1 for the regression task. The inputs of HybridGCN consist of nodes and edges, specifically, we extract six sets of protein features as the node features *V*, including five traditional protein features relevant to different protein properties and one powerful deep feature extracted from the zero-shot deep learning network ESM-1v [38].

The node features we selected include the Blosum62 [39] feature ($F_{blosum}$), the physicochemical property feature AAPHY7 [40] ($F_{aap}$), the position-specific scoring matrix PSSM [41] ($F_{pssm}$), the Hidden Markov matrix HMM [42] ($F_{hmm}$), and the predicted structural feature SPIDER3 [43] ($F_{spider}$). Blosum62 is a 20 $\times$ 20 matrix for substitutions between 20 standard amino acid types according to alignments of homologous protein sequences. AAPHY7 is a set of 7 physicochemical properties for amino acid types. Both PSSM and HMM are evolutionary features that may contain information related to protein properties such as the solubility of proteins. SPIDER3 is a structural feature predicted from the structural predictor SPIDER3, which may be related to the solubility of proteins. ESM-1v is a powerful deep learning feature extracted from the zero-shot protein language model. We will describe it in detail in the next subsection. The summary of the edge features can be found in Table 1.

**Table 1:** Six types of features and dimensions

Names	Properties	Dimensions
ESM-1v	Protein Function and Structure	20
BLOSUM62	Block Substitution Matrix	20
AAPHY7	Physicochemical Properties	7
PSSM	Position-specific Scoring Matrix	20
HMM	Hidden Markov Matrix	30
SPIDER3	Structural Properties Predicted by SPIDER3	14

For the edge feature, we select the protein contact map predicted by SPOT-Contact [44]. The contact map represents 2D structural features and contains all the possibilities to form contacts between all residue pairs in one protein. In contrast to the previous GCN-based solubility prediction network GraphSOL [19], we leverage a feature extracted from the zero-shot deep learning protein language model ESM-1v to enhance the node features. Moreover, we propose a novel adaptive feature re-weighting module to explore the interactions between different features and extract the most informative ones. We will describe these two novel components as follows.

### Zero-shot feature learning model ESM-1v

ESM-1v, a 650 M parameter transformer-based protein language model, is pre-trained on large and diverse protein sequence databases containing 98 million protein sequences from across the tree of life. It is trained with the masked language modeling objective to predict the probability that an amino acid occurs at a position in a protein given the surrounding context. After pre-training, the ESM-1v model transfers without supervision from experimental data, to predict the effects of sequence mutations on protein function. Extensive experimental results show ESM-1v develops an understanding of sequences that reflect the protein function and structure. Hence, the output probabilities of ESM-1v model are used to predict the effects of sequence mutations on protein function [38].

Considering the capability of the ESM-1v model for learning general information relevant to protein function and structure, we exploit it as a zero-shot feature learning network. As shown in Fig. 1, we directly extract features of the protein solubility datasets using the ESM-1v model without further training. The ESM-1v features are taken as the input of the following protein solubility prediction network. Specifically, we extract the ESM-1v model’s output probabilities as informative deep features to assist the protein solubility prediction task. Let $P \in {\mathbb {R}}^L$ denotes an input protein sequence of length *L*, we extract the ESM-1v feature using the ESM-1v mapping:

1 $$F_{esm}=ESM_{1v}(P)$$

where $F_{esm}$ is the output feature of ESM-1v model, and $ESM_{1v}(.)$ is the ESM-1v mapping function. The model ESM-1v output probabilities of 25 amino acid classes, in practice, we extract the output probabilities of 20 commonly used amino acid classes purely as the final features, i.e., $F_{esm}\in {\mathbb {R}}^{L\times 20}$.

## Adaptive Feature Re-weighting (AFR)

The adaptive feature re-weighting (AFR) module *R*, taking multiple features as inputs, learns to encode the most informative information of each feature into a hybrid re-weighting feature and adjust the contribution of each feature according to its contribution to the solubility prediction task. With the re-weighting module, the features informative for solubility prediction would be excited and thus improve prediction performance. Formally, the AFR module consists of three stages: hybrid feature construction, feature weight inference, and feature re-weighting.

### Hybrid feature (H) construction

Formally, Let *P* denote an input protein sequence, and $H\in {\mathbb {R}}^{L\times C}$ denotes its corresponding input feature, here, *L* is the length of the protein sequence and *C* is the channel number of the feature. We extract 6 types of features and stack them into a hybrid input feature *H* as follows:

2 $$H=[F_{esm}, F_{blosum}, F_{aap}, F_{pssm}, F_{hmm}, F_{spider}]$$

Where $F_{esm}$, $F_{blosum}$, $F_{aap}$, $F_{pssm}$, $F_{hmm}$ and $F_{spider}$ denote the ESM feature, the Blosum62 feature, the PSSM feature, the AAPHY7 feature, the HHM feature and the SPIDER3 feature, respectively.

The feature re-weighting module ${\textbf{AFR}}$ takes the hybrid feature as input and encodes the hybrid feature into a re-weighted feature representation *R* using the channel-specific weight *W*

3 $$R= {\textbf{AFR}}(H, W)$$

### The Feature Weight (${\textbf{W}}$) Inference

The hybrid feature *H* is obtained by stacking a series of channels, which can be re-written as:

4 $$\begin{aligned}H & =[F_{esm}, F_{blosum}, F_{aap}, F_{pssm}, F_{hmm}, F_{spider}] \\ & =[H_{1}, H_{2},..., H_{c},..., H_{C-1}, H_{C}] \end{aligned}$$

$H_c$ indicates the *C*-th channel of the hybrid feature.

We first extract the global feature of each channel using the global average pooling transformation $\mathbf {F_{gap}(.):H \rightarrow G}, {\textbf{H}} \in {\mathbb {R}}^{L\times C}, {\textbf{G}} \in {\mathbb {R}}^{1\times C}$.

5 $$G={\textbf{F}_{\textbf{gap}}}(H)=[F_{gap}(H_1),..., F_{gap}(H_c),..., F_{gap}(H_C)]$$

6 $$G_c={\textbf{F}_{\textbf{gap}}}(H_c)=\frac{1}{L}\sum _{i=1}^L H_{c}^i$$

where $G_c$ is the global feature of the *c*-th channel, and $H_c^i$ is the *i*-th element in the *c*-th channel of the hybrid feature.

Then, we extract the nonlinear interaction features between channels from the global features. The nonlinear interaction features are captured by a convolution function and a ReLU function, among which the convolution function extracts the linear interaction features and the ReLU function and adds nonlinearity into the interaction features. Let $V=[v^1, v^2,..., v^{C'}]$ denotes the learned set of convolution filter kernels, where $v^{c'} \in {\mathbb {R}}^{1 \times C}$ refers to the parameters of the *c*-th filter. To match the length of the input and output of the convolution function, we use *C* convolution filters, i.e., $C'=C$. The linear interaction feature $O=[o_1, o_2,..., o_{C'}]$ can be obtained as

7 $$o_{c'}=v^{c'}*G=\sum _{c=1}^{C}v_{c}^{c'}*G_c$$

Here $*$ denotes the convolution operator, $v^{c'}=[v^{c'}_1,v^{c'}_2,..., v^{c'}_C] \in {\mathbb {R}}^{1 \times C}$ and $G=[G_1,G_2,..., G_C] \in {\mathbb {R}}^{1 \times C}$. Next, we adopt the ReLU function to introduce nonlinearity into the interaction features, enabling the interaction features to capture more complex and realistic interaction information. The nonlinear interaction features $P=[P_1,P_2,..., P_C] \in {\mathbb {R}}^{1 \times C}$ can be formulated as

8 $$P={\textbf{F}}_{nl}(O)=max(0, O)$$

Finally, we derive the feature channel weight with the global information and interaction information. Specifically, we choose the sigmoid activation function to derive the weight of each channel $W_c$.

9 $$W_c=\textbf{F}_{sm}(P_c) =\frac{1}{1+e^{-P_c}}$$

Here $W=[W_1, W_2,...,W_C]$ is the final feature channel weight. The sigmoid function outputs a value between 0 and 1 for each channel, describing the importance of each channel. A value of zero means that the feature channel is meaningless to the solubility prediction task, while a value of one indicates that the feature channel is the most informative one.

### Feature re-weighting

Once the feature channel weight is achieved, we then perform the feature re-weighting as

10 $$R= H \otimes W$$

where *R* indicates the re-weighted hybrid feature, and $\otimes$ indicates channel-wise multiplication. The channel-specific coefficient *W* highlights more informative and relevant feature channels to predict the solubility, hence, the re-weighted feature *R* is able to capture solubility favorable representations and improve the performance of solubility prediction.

## Experimental setup

To demonstrate the effectiveness of the proposed method, we conduct comprehensive evaluations on open datasets. In this section, we first introduce the experimental datasets and evaluation metrics. Then, we describe the implementation details.

### Datasets

**eSOL dataset** [45]. For the model training, we utilized the eSOL dataset obtained from a previous study [45]. Solubility in this dataset was defined as the ratio of the supernatant fraction to the total fraction in physiochemical experiments referred to as PURE [46]. For fair comparisons, we used the same dataset setting as GraphSol [19]. The final dataset encompassed a total of 2,737 protein sequences, 75% of the samples were randomly selected as training data, while the remaining 25% were designated as independent test data. Most of our experiments were conducted using this dataset, as it has more samples for detailed investigation.

**S. cerevisiae dataset** [47]. To comprehensively evaluate the proposed HybridGCN, we selected the S. cerevisiae dataset collected by [47] as an external independent test. This dataset has fewer samples with 108 proteins and their corresponding 3D structures. The solubility was also measured by the cell-free expression called PURE [46].

### Evaluation metrics

**Regression evaluation metrics.** Our focus in our study is to predicate every value of protein solubility. Thus we frame protein solubility prediction as a regression task, aiming to predict specific solubility values for proteins rather than classifying them into soluble or insoluble categories. Following the approach of GraphSOL, we employ the root mean squared error (RMSE) as a loss value, which serves as one of our evaluation metrics for the final trained deep model. Additionally, we utilize the coefficient of determination (R2) to assess the performance of our models and optimize the hyperparameters.

**Classification evaluation metrics.** The majority of previous studies have formulated protein solubility prediction as a classification task, involving the classification of proteins into soluble or insoluble categories. In line with this approach, we also segregated all proteins using a threshold of 0.5. Specifically, if the predicted or true solubility value of a protein fell below the threshold of 0.5, it was classified as insoluble; otherwise, it was considered soluble. As the task is classification-oriented, we employed several classification metrics to evaluate the performance of the prediction model. These metrics include the Area under the ROC Curve (AUC), accuracy, precision, recall, and F1 score, defined as follows:

11 $$Precision = \frac{TP}{TP+FP}$$

12 $$Recall = \frac{TP}{TP+FN}$$

13 $$F_1 = 2\times \frac{Precison \times Recall}{Precision + Recall}$$

### Cross-validation and independent test

To ensure robustness and generalizability, we perform 5-fold cross-validation on the training dataset. Specifically, the proteins in the training dataset are divided into five separate folds. In each round, four folds are utilized for training a model, which is subsequently evaluated on the remaining one-fold. This process is repeated five times, and the performances of the five predictions are averaged to obtain the validation performance. To mitigate fluctuations resulting from random splitting, we used five different random seeds and averaged the final performances. The validation phase is crucial for hyperparameter optimization. After fine-tuning the optimal hyperparameters, a model was trained using the entire training dataset and independently tested on two separate test datasets.

### Implementation details

Our detection framework is implemented using the Keras open-source machine learning framework. All experiments are conducted on a server equipped with an Intel Xeon CPU @ 2.40GHz and a single Nvidia Tesla P100 GPU with 16 GB of memory.

## Results and discussion

In this section, we present and discuss the experimental results and findings. HybridGCN, built upon the GCN framework, incorporates two novel components, namely the AFR module and the ESM-1v feature, into a standard GCN. We begin by conducting ablation experiments to assess the contributions of the AFR module and the ESM-1v feature to the overall performance of HybridGCN. Subsequently, we analyze the individual influences of each feature, including ESM-1v, Blosum62, AAPHY7, PSSM, HMM, and SPIDER3, on HybridGCN. Finally, we compare our method against several SOTA protein solubility prediction methods on the eSOL dataset and the S. cerevisiae dataset.

### Ablation study of the AFR module and ESM-1v feature on eSOL

**Table 2:** Ablation studies of the AFR module and ESM-1v feature on the 5-fold cross-validation set of eSOL

Methods	RMSE	$R^2$	Accuracy	Precision	Recall	F1	AUC
GCN	0.231	0.483	0.779	0.775	0.693	0.732	0.866
ESM+GCN	0.229	0.493	0.782	0.779	0.713	0.740	0.870
AFR+GCN	0.230	0.488	0.781	0.778	0.715	0.742	0.871
ESM+AFR+GCN (HybridGCN)	**0.227**	**0.497**	**0.783**	**0.780**	**0.722**	**0.749**	**0.876**

The best values are marked in bold

The concept of an ablation study arises when specific components of a model are removed to gain a better understanding of their contribution to the overall model performance. In our ablation study, we individually remove the AFR module and the ESM-1v feature from HybridGCN, resulting in two distinct models: the ESM+GCN model and the AFR+GCN model. We compare the performance of these models with the overall HybridGCN (ESM+AFR+GCN) model and the standard GCN model. The performances on the 5-fold cross-validation set of eSOL are reported in Table 2.

The inclusion of the ESM-1v feature in GCN leads to significantly improved $R^2$ (0.493) and Recall (0.713) compared to GCN alone. The notable performance gains primarily stem from the utilization of the ESM-1v feature, which acts as a powerful zero-shot learning feature that has assimilated information pertaining to protein structure and function. This further reinforces the notion that protein solubility is closely linked to protein structure and function. The AFR module also enhances the performance of GCN across all evaluation metrics. These findings indicate that not all individual features are equally important, and a superior composite feature is learned through the AFR module, which effectively highlights the most informative features related to protein solubility.

**Fig. 2:**
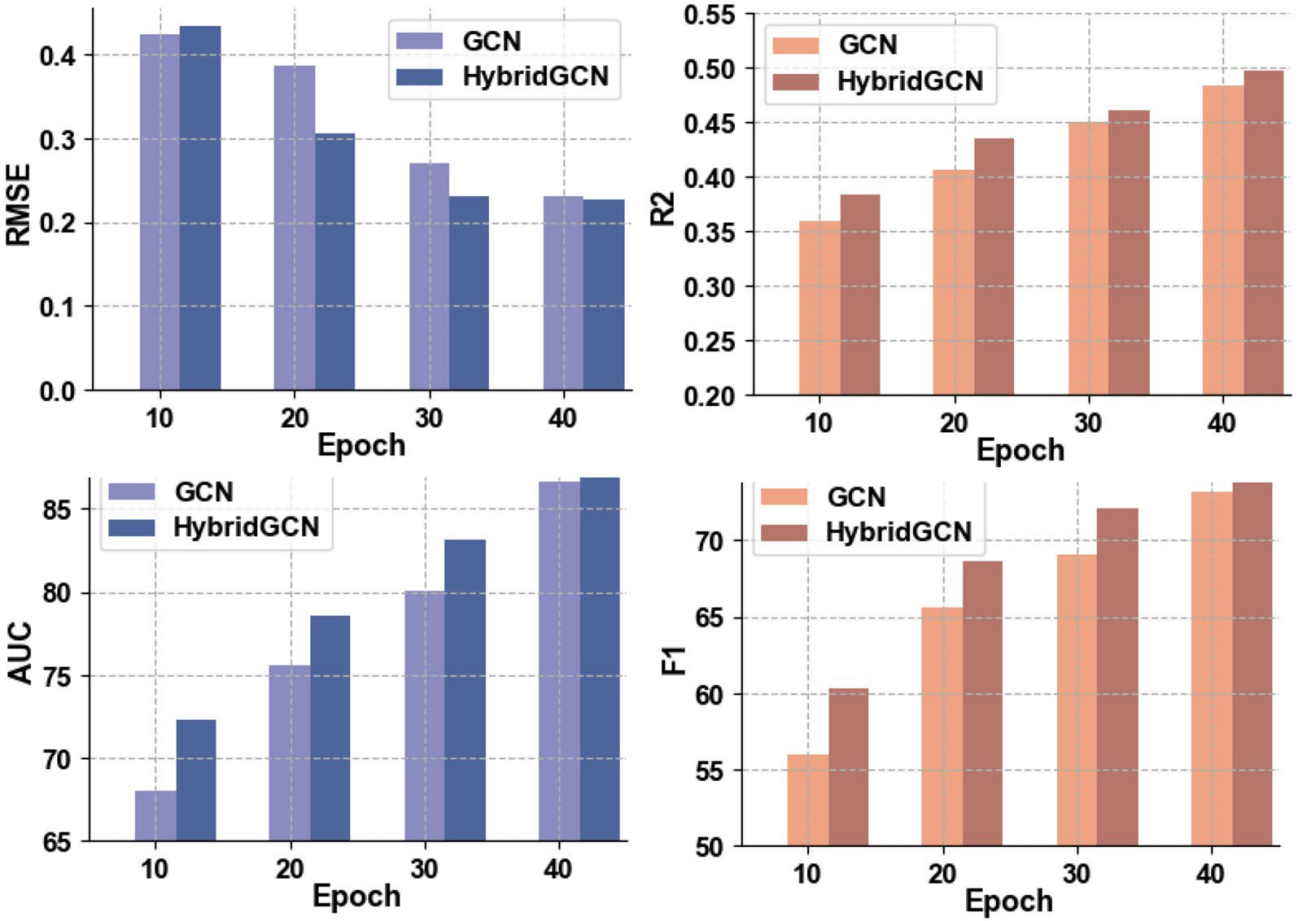
The performance HybridGCN and GCN at different training epochs

Figure 2 illustrates the consistent outperformance of HybridGCN over GCN at different training epochs and across various evaluation metrics. The overall HybridGCN exhibits the best performance among the four settings in terms of both regression metrics and classification metrics. The observed performance improvements can be attributed to the contributions of both the ESM-1v feature and the AFR module. These results underscore the importance of a well-designed feature engineering strategy in the protein solubility prediction task.

### Analysis of the influences of individual features on eSOL

As feature engineering plays a crucial role in protein solubility prediction, it is important to identify the features that have the most significant impact on the task. We designed two groups of experiments: (1) using individual features as inputs to GCN, where only one feature is utilized for the solubility prediction without applying the AFR module; (2) removing individual features from the overall HybridGCN, i.e., conducting ablation experiments.

**Table 3:** The performance ( $R^2$ ) of HybirdGCN with individual features or ablated features. We present $R^2$ on both the 5-fold cross-validation set and the test set of eSOL

Individual feature	Validation	Test	Ablated feature	Validation	Test
-	-	-	-None (HybridGCN)	**0.495± 0.012**	**0.497**
ESM	**0.372± 0.012**	**0.365**	-ESM	0.476± 0.013	0.488
HMM	0.337± 0.015	0.331	-HMM	0.478± 0.018	0.490
PSSM	0.333± 0.012	0.332	-PSSM	0.479± 0.015	0.491
BLOSUM	0.329± 0.016	0.317	-BLOSUM	0.485± 0.015	0.490
SPIDER3	0.293± 0.014	0.289	-SPIDER3	0.490± 0.012	0.493
AAPHY7	0.231± 0.019	0.227	-AAPHY7	0.488± 0.014	0.490

The best values are marked in bold

“-None” indicates the complete HybridGCN without feature ablation

The performances (measured by $R^2$) of HybridGCN with individual features or ablated features are presented in Table 3. From the results of the individual features, we observed that the ESM-1v feature had the highest importance for the solubility prediction task, as HybridGCN with the ESM-1v feature achieved the highest $R^2$ values on both the validation set (0.372 ± 0.012) and test set (0.365). The HMM feature and PSSM feature demonstrated similar $R^2$ values on the validation set (0.337 ± 0.015 for HMM and 0.333 ± 0.012 for PSSM) and test set (0.331 for HMM and 0.332 for PSSM). It is noteworthy that PSSM and HMM capture evolutionary information, which is relevant to protein solubility. On the other hand, AAPHY7 exhibited the lowest $R^2$ values on the validation set (0.231 ± 0.019) and test set (0.227) due to its smaller dimensionality compared to other features.

Regarding the ablation experiments, removing the ESM feature results in the largest performance drop, reducing $R^2$ values from 0.495 ± 0.012 to 0.476 ± 0.013 on the validation set and from 0.497 to 0.488 on the test set. These findings further confirm the high importance of the ESM feature for protein solubility prediction. Conversely, removing the SPIDER3 feature led to the smallest drop, with $R^2$ values decreasing from 0.495 ± 0.012 to 0.490 ± 0.012 on the validation set and from 0.497 to 0.493 on the test set. This can be attributed to the fact that SPIDER3 is not the sole feature capturing structural information, as the ESM-1v feature also encodes structural information of the protein sequences. Moreover, the structural information contained in the ESM-1v feature is more closely related to protein solubility than the information preserved in SPIDER3.

**Table 4:** The $R^2$ of the model merging the ESM feature with other individual features

Individual Feature	Validation	Test
ESM	0.372± 0.012	0.365
ESM+HMM	**0.423± 0.011**	**0.420**
ESM+PSSM	0.415± 0.013	0.411
ESM+BLOSUM	0.398± 0.012	0.394
ESM+AAPHY7	0.388± 0.014	0.382
ESM+SPIDER3	0.392± 0.019	0.386

The best values are marked in bold

**Fig. 3:**
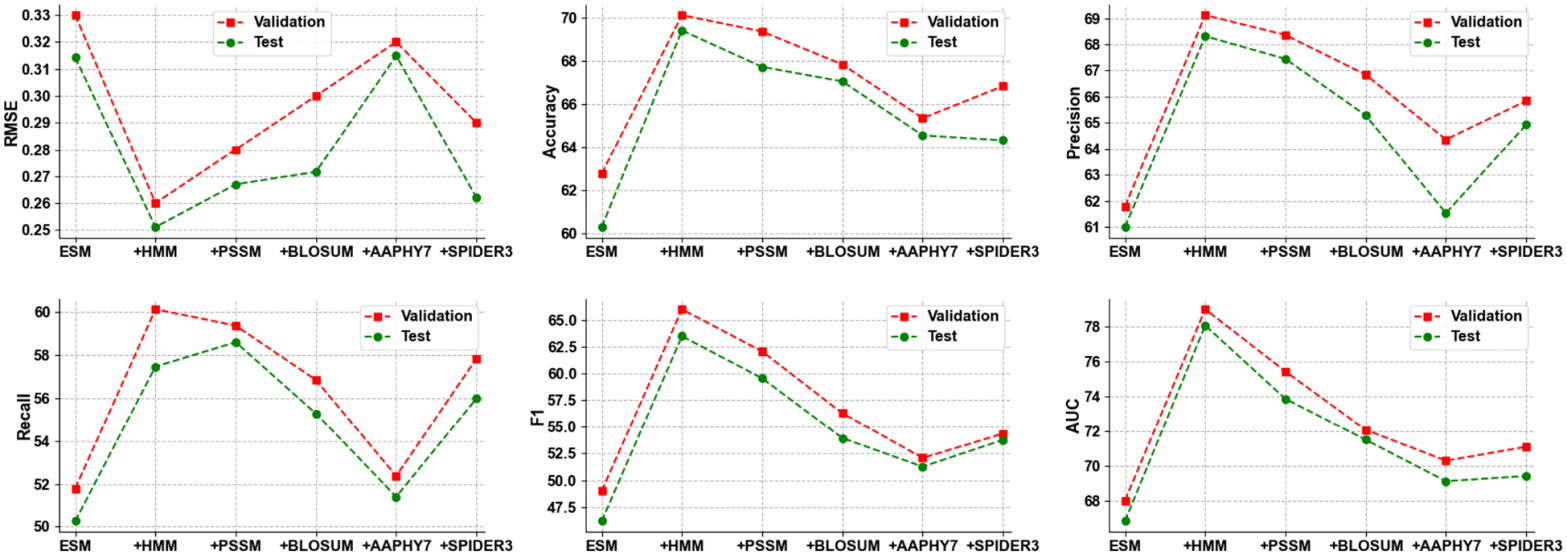
The performance of combinations of ESM-1v and other individual features. HMM, containing rich evolutionary information, is the best complementary feature to ESM-1v

**Table 5:** Performance comparisons with SOTA methods

Methods	RMSE	$R^2$	Accuracy	Precision	Recall	F1	AUC
K-nearest Neighbor	0.284	0.214	0.691	0.737	0.486	0.586	0.776
Linear Regression	0.280	0.240	0.707	0.685	0.642	0.663	0.777
Random Forest	0.255	0.370	0.760	0.750	0.690	0.729	0.825
XGboost	0.252	0.385	0.756	0.748	0.690	0.718	0.829
LSTM	0.236	0.458	0.765	0.748	0.677	0.730	0.855
SVM	0.246	0.411	0.761	0.763	0.684	0.721	0.842
ProteinSol	0.253	0.376	0.714	0.689	0.688	0.693	0.808
DeepSol	0.241	0.434	0.763	0.771	0.738	0.695	0.845
ProGAN	0.237	0.442	0.763	0.770	0.676	0.720	0.853
SeqVec	0.236	0.458	0.767	0.754	0.715	0.734	0.858
TAPE	0.235	0.461	0.764	0.774	0.710	0.730	0.856
NetSolP	0.240	0.449	0.760	0.768	0.716	0.722	0.833
GraphSOLSingle	0.231	0.483	0.779	0.775	0.693	0.732	0.866
GraphSOLEnsemble	0.227	0.501	0.782	0.790	0.702	0.743	0.873
OursSingle	0.227	0.497	0.783	0.780	0.722	0.749	0.876
OursEnsemble	**0.226**	**0.510**	**0.801**	**0.816**	**0.729**	**0.764**	**0.886**

The best values are marked in bold

To investigate the complementary nature of the features with the ESM feature, we conducted further experiments by combining the ESM-1v feature with other individual features. Table 4 and Fig. 3 demonstrate the performance of combinations of the ESM-1v feature with other individual features. From these results, we observed that the HMM feature is the best complementary feature to the ESM-1v feature, while the PSSM feature is the second best complementary feature. These findings indicate that the evolutionary information captured by HMM and PSSM is the most beneficial complement to the structural information provided by the ESM-1v feature for the protein solubility prediction task.

### Comparisons with SOTA methods on the eSOL dataset

Our proposed HybridGCN model is compared with several SOTA protein solubility prediction methods on the eSOL dataset, including GraphSoLEnsemble [19], GraphSoLSingle [19], NetSolP [31], DeepSoL [9], SeqVec [7], TAPE [30], and ProGAN [29]. Additionally, we compare HybridGCN with several classical machine learning models, including Long Short-term Memory (LSTM), SVM, K-nearest Neighbor (KNN), Linear Regression (LR), Random Forest (RF), and XGboost.

Quantitative results of the different comparison methods are presented in Table 5. Among the various comparative methods evaluated, GraphSoLEnsemble and GraphSoLSingle exhibit good performance, as evidenced by higher $R^2$ values on the eSOL dataset. This notable achievement can be attributed to the utilization of GCN as the underlying architecture in network models, showcasing the remarkable analytical capabilities of GCN in handling graph-structured data. Within the spectrum of models assessed, the OursEnsemble model emerges as the top-performing model, surpassing the SOTA protein solubility predictor GraphSoLEnsemble [19]. The OursEnsemble model demonstrates superiority by achieving a margin of 1.9%, 2.6%, 2.7%, and 2.1% in accuracy, precision, recall, and F1 score, respectively. This outstanding performance can be attributed to the incorporation of two key components within our HybridGCN framework: the AFR module and the ESM-1v feature.

The AFR module, serving as an optimized feature fusion mechanism, plays a pivotal role in adjusting the importance of features based on their relevance to solubility prediction. This strategic adaptation contributes to the model’s heightened discriminative capabilities. Additionally, the inclusion of the ESM-1v feature, a potent deep learning feature derived from protein sequences, proves instrumental in capturing intricate information pertaining to protein structure and function. Leveraging large language models, the ESM-1v feature significantly enhances the overall solubility prediction performance. In summary, our HybridGCN model, enriched by the AFR module and the ESM-1v feature, establishes a new benchmark in protein solubility prediction, outperforming existing SOTA predictors and showcasing the efficacy of the proposed enhancements in feature fusion and deep learning representation.

SeqVec and TAPE are transfer learning frameworks for solubility prediction that make use of deep features or embeddings taken from pre-trained deep networks as inputs for the solubility prediction task. However, their performance is not as good as HybridGCN, which is due to the lack of traditional features, such as evolutionary features, that could supplement the deep features. DeepSoL incorporates additional biological and structural features to improve the utility of deep features, but its performance is limited by the use of basic CNN as the network backbone. Although it includes features from multiple sources, it only relies on simple concatenation operations to combine these features, which may not effectively select and enhance the most informative features for solubility prediction. In comparison, HybridGCN uses GCN as the network backbone, allowing direct processing of graph structures and taking advantage of the structural information of proteins. Among the classical machine learning models, LSTM performs the best, which is not surprising considering its suitability for processing sequential data such as protein sequences. Nevertheless, our HybridGCN consistently outperforms all other models across all metrics due to the introduction of the AFR module and the ESM-1v feature.

### Comparisons with SOTA methods on the S. cerevisiae dataset

**Table 6:** Performance comparisons with SOTA methods on the S. cerevisiae dataset

Solubility predictors	$R^2$
ProGAN	0.084
DeepSol	0.090
Protein-Sol	0.281
ccSol	0.302
GraphSOLSingle	0.358
GraphSOLEnsemble	0.372
OursSingle	0.378
OursEnsemble	**0.390**

The best value is marked in bold

We also compared our HybridGCN with other top performance methods on the S. cerevisiae dataset, including ProGAN [29], DeepSol [9], ProteinSol [48], ccSol [20], and GraphSol [19]. Specifically, we train all methods on the eSOl training dataset and test them on the S. cerevisiae dataset, examining the generalization ability of different models.

The results of different methods on the S. cerevisiae dataset are presented in Table 6, from which we find OursEnsemble achieves the best $R^2$ (0.390) among the comparison methods, showing the advantage of HybridGCN over other methods in modelling feature relationships. It is also worth noting that OurSingle ($R^2$=0.378) outperforms GraphSolEnsemble ($R^2$=0.372) on the S. cerevisiae dataset, even though the performance gain is small, our single model is more efficient than the ensemble model during inference.

## Conclusions

This paper presents HybridGCN, a novel graph convolutional network model that combines deep learning features with classic solubility-related features to improve the accuracy of protein solubility prediction. This model takes into account structural and biological features of protein sequences, as well as a deep learning feature extracted from high-capacity large language models, to enhance prediction performance. Our analysis can identify the interplay between deep features and classic biological features, where certain classic features complement the deep features in the solubility prediction task. To further improve the prediction task, the ESM-1v feature, a zero-shot learning feature, was introduced to capture comprehensive and relevant information on protein functions and structures. Additionally, an adaptive feature re-weighting module was proposed to explore feature interactions and enhance the most informative features for solubility prediction. Ablation experiments and comparisons demonstrate the efficacy of the ESM-1v feature and the AFR module. HybridGCN achieved SOTA performance on the publicly available eSOL dataset.

The utilization of sophisticated deep learning features with classical biological features manifests a notable enhancement in the predictive performance of protein solubility. Crucially, the discernment of feature importance through feature re-weighting emerges as a noteworthy aspect, holding promise for broader applications in the realm of protein engineering. This strategic identification of pivotal features not only refines the solubility prediction task but also presents avenues for addressing diverse inquiries within the field. Given the escalating computational costs associated with an expanding repertoire of features in machine learning models, the role of feature re-weighting becomes paramount. It assumes a critical function in the selection of target features, while concurrently removing less salient features, tailored to the aims of specific tasks. This discerning feature management proves instrumental in mitigating computational overhead, thereby optimizing the efficiency of predictive models.

An inherent limitation of our HybridGCN lies in its departure from an end-to-end deep learning framework. The necessity to rely on external feature extractors for the extraction of node and edge features introduces additional time expenses. However, this procedural choice provides the advantage of using more advanced large protein language models for feature engineering [49–51], underscoring a trade-off between computational efficiency and leveraging state-of-the-art language models. In future endeavors, we aspire to delve into alternative deep learning models for both feature extraction and fusion [35]. By exploring and integrating advanced methodologies, we aim to further refine the efficiency and scope of our predictive models, paving the way for enhanced insights into protein solubility and related applications.

## Data Availability

The data is from a public dataset which is available at https://github.com/jcchan23/GraphSol/tree/master/Data. Our source code will be made public at https://github.com/IanDragon once accepted.
